# Efficacy and safety of hyperbaric oxygen therapy in acute ischaemic stroke: a systematic review and meta-analysis

**DOI:** 10.1186/s12883-024-03555-w

**Published:** 2024-02-03

**Authors:** Xuezheng Li, Lijun Lu, Yu Min, Xuefeng Fu, Kaifeng Guo, Wen Yang, Hao Li, Haoming Xu, Hua Guo, Zhen Huang

**Affiliations:** 1 Postgraduate cultivation base of Guangzhou University of Chinese Medicine, Panyu Central Hospital, Guangzhou Guangdong, China; 2 South China Normal University, Guangzhou Guangdong, China; 3 Department of Rehabilitation Medicine, Guangzhou Panyu Central Hospital, No. 8, Fuyu East Road, South Bridge Street, Panyu District, Guangzhou, China

**Keywords:** Acute ischaemic stroke, Hyperbaric oxygen, Meta-analysis, Systematic review, Randomized controlled trials, Rehabilitation

## Abstract

**Objective:**

This study aims to evaluate the efficacy and safety of adjunctive hyperbaric oxygen therapy (HBOT) in acute ischaemic stroke (AIS) based on existing evidence.

**Methods:**

We conducted a comprehensive search through April 15, 2023, of seven major databases for randomized controlled trials (RCTs) comparing adjunctive hyperbaric HBOT with non-HBOT (no HBOT or sham HBOT) treatments for AIS. Data extraction and assessment were independently performed by two researchers. The quality of included studies was evaluated using the tool provided by the Cochrane Collaboration. Meta-analysis was conducted using Rev Man 5.3.

**Results:**

A total of 8 studies involving 493 patients were included. The meta-analysis showed no statistically significant differences between HBOT and the control group in terms of NIHSS score (MD = -1.41, 95%CI = -7.41 to 4.58), Barthel index (MD = 8.85, 95%CI = -5.84 to 23.54), TNF-α (MD = -5.78, 95%CI = -19.93 to 8.36), sICAM (MD = -308.47, 95%CI = -844.13 to 13227.19), sVCAM (MD = -122.84, 95%CI = -728.26 to 482.58), sE-selectin (MD = 0.11, 95%CI = -21.86 to 22.08), CRP (MD = -5.76, 95%CI = -15.02 to 3.51), adverse event incidence within ≤ 6 months of follow-up (OR = 0.98, 95%CI = 0.25 to 3.79). However, HBOT showed significant improvement in modified Rankin score (MD = 0.10, 95%CI = 0.03 to 0.17), and adverse event incidence at the end of treatment (OR = 0.42, 95%CI = 0.19 to 0.94) compared to the control group.

**Conclusion:**

While our findings do not support the routine use of HBOT for improving clinical outcomes in AIS, further research is needed to explore its potential efficacy within specific therapeutic windows and for different cerebral occlusion scenarios. Therefore, the possibility of HBOT offering clinical benefits for AIS cannot be entirely ruled out.

**Supplementary Information:**

The online version contains supplementary material available at 10.1186/s12883-024-03555-w.

## Introduction

Stroke is a severe cerebrovascular disease recognized by the World Health Organization as one of the leading healthcare problems worldwide [1]. Acute ischaemic stroke (AIS) is the predominant subtype, accounting for approximately 85% of all strokes [2, 3]. It results from the blockage of cerebral blood vessels by thrombi or other emboli, leading to insufficient oxygen and nutrient supply and subsequent necrosis of brain tissue [4]. AIS ranks as the second leading cause of death globally [5]. Even for those who survive, stroke often leads to various complications and sequelae, including motor impairments, cognitive deficits, and speech disorders. The high mortality rate, disability rate, and significant economic burden on patients, families, and society [6] make it imperative to explore effective treatment methods for AIS.

The primary effective treatments for AIS are thrombolysis and thrombectomy [7, 8]. Administering intravenous thrombolysis within 4.5 h can benefit patients, with earlier treatment yielding greater benefits [9]. Compared to thrombolysis, mechanical thrombectomy has a longer therapeutic window. A previous study indicated benefits from mechanical thrombectomy within 24 h after stroke onset [10]. However, strict time windows and indications limit the application of these interventions. In such cases, conventional medical treatment is generally used, such as aspirin and statins for preventing further platelet aggregation and limiting infarct expansion. Additionally, non-pharmacological, non-invasive therapies like oxygen therapy, acupuncture, and electrical stimulation are sometimes used alongside conventional methods and are gaining increasing attention. Studies increasingly suggest these therapies may offer potential therapeutic benefits for AIS patients [11, 12].

Local neuronal ischemic hypoxic necrosis is generally considered the primary cause of brain tissue damage in AIS [13]. Hyperbaric oxygen therapy (HBOT) is commonly used as an adjunctive treatment for AIS [14], aiming to improve tissue oxygenation and restore neuronal activity in metabolically compromised regions. A study showed that HBOT can increase arterial oxygen tension by raising dissolved oxygen levels in plasma, thereby enhancing tissue oxygenation [15]. Furthermore, animal research has demonstrated that HBOT can stimulate the expression of nutrient and neurotrophic factors in rats with IS, promoting the homing of bone marrow-derived mesenchymal stem cells to the ischemic brain area and enhancing cellular repair [16]. However, the application of HBOT in AIS remains controversial [17]. Some studies indicate that HBOT exerts neuroprotective effects through multiple pathways and targets [18, 19]. However, other studies suggest that HBOT does not appear to improve outcomes in AIS patients [14, 17]. Previous meta-analyses have shown no substantial evidence supporting the use of HBOT during the acute phase of IS to improve clinical outcomes [14, 20]. However, these studies did not completely rule out the possibility of clinical benefits of HBOT, and many scale data and biomarker data were not included in these analyses. Additionally, subsequent clinical studies on HBOT for IS treatment have emerged, demonstrating positive therapeutic outcomes.

Therefore, we aim to conduct a meta-analysis of clinical trials of adjunctive HBOT for AIS, focusing on patients who did not receive thrombolysis/thrombectomy. This research will clarify the therapeutic role of HBOT for AIS by explaining these conflicting results and informing clinical management.

## Methods

### Study registration

The protocol for this systematic review was registered on the PROSPERO platform on May 19, 2023, with the registration ID CRD 42,023,424,572, and this study was implemented following the PRISMA statement [21].

### Search strategy

A comprehensive search was conducted in seven databases, including PubMed, Embase, Web of Science, Cochrane Library, ClinicalTrials.gov, EudraCT, and WHO International Clinical Trial Registry Platform. The search covered all English-language literature from the inception of each database up to April 15, 2023. The search terms encompassed “ischemic stroke” and “hyperbaric oxygen”. The search terms included “Ischaemic Stroke,” “Cryptogenic Embolism Stroke,” “Wake up Stroke,” “Hyperbaric Oxygenation,” and “Hyperbaric Oxygen Therapy.” Search terms and strategies were adjusted and refined based on the requirements of each database to ensure a thorough search across all databases. Detailed search strategies are provided in Appendix 1.

### Inclusion and exclusion criteria

#### Study type

All randomized controlled trials (RCTs) of HBOT treatment in AIS were included, while non-randomized studies, observational studies, animal experiments, qualitative studies, case reports, expert opinions, and letters were excluded.

#### Participants

The study population included patients with AIS of any gender or age. All patients were diagnosed with ischemic stroke using CT or MRI, and those with hemorrhagic stroke were excluded. Additionally, all study participants had no clear contraindications for oxygen therapy.

#### Interventions

The experiment group received adjunctive HBOT combined with conventional medical treatment. In some cases, HBOT may be combined with other rehabilitation methods like acupuncture or rehabilitation training, but these methods are identical for both the experimental and control groups (apart from blinding strategies like sham HBOT). Trials involving patients who received thrombolysis/thrombectomy were excluded.

#### Outcome measures

To assess the effects of HBOT on AIS, the following outcomes were considered as primary outcome measures: (1) NIHSS score; (2) Barthel index; (3) modified Rankin Scale score; (4) TNF-α; (5) sICAM; (6) sVCAM; (7) sE-selectin; (8) CRP. Secondary outcome measures included the number and severity of adverse events.

### Data extraction

After the literature search, titles and abstracts of the retrieved articles were imported into EndNote 20 software. After removing duplicate records, two reviewers (Xuezheng Li and Lijun Lu) independently read the titles and abstracts of the articles to exclude studies that clearly did not meet the inclusion criteria. Subsequently, a full-text screening was conducted to determine the eligibility of potentially relevant studies. For eligible articles, data extraction was independently performed by two reviewers. Extracted data consisted of general information (e.g., first author, publication year, sample size, randomization method, group allocation), participant characteristics (e.g., gender, age, duration of disease), intervention details (e.g., frequency of therapy, hyperbaric oxygen parameters, start time), outcome data, follow-up results and time, and adverse event information. Any discrepancies were resolved by a third reviewer (Yu Min). If specific data were not provided but only presented in graphical form, Engauge Digitizer 10.8 software was used for data extraction from the graphs.

### Quality assessment

Each included study was assessed for risk of bias using the Cochrane Collaboration’s tool [22]. The items in the tool were divided into seven domains: (A) random sequence generation; (B) allocation concealment; (C) blinding of participants and personnel; (D) blinding of outcome assessment; (E) incomplete outcome data; (F) selective reporting; (G) other biases. Each domain of the included studies was rated as low, high, or unclear risk of bias, and classified as “yes” (low bias for items a-e, high bias for items f-g), “no” (high bias for items a-e, low bias for items f-g), or “unclear” (lack of relevant information or uncertain bias). Two reviewers (Xuefeng Fu and Hao Li) independently performed the assessment, and any discrepancies were resolved by a third reviewer (Wen Yang).

### Statistical analysis

Statistical analysis of the included RCTs was conducted using Review Manager 5.3 software. The data included both dichotomous variables and continuous variables. For dichotomous variables, odds ratios (ORs) with 95% confidence intervals (CIs) were calculated, while mean differences (MDs) with 95%CIs were calculated for continuous variables. Heterogeneity among the studies was assessed using the I^2^ test. If I^2^ < 50%, a fixed-effects model was used to analyze the data. Conversely, if I^2^ ≥ 50%, a random-effects model was employed. Publication bias was examined using a funnel plot.

## Results

### Literature screening process and results

**Fig. 1:**
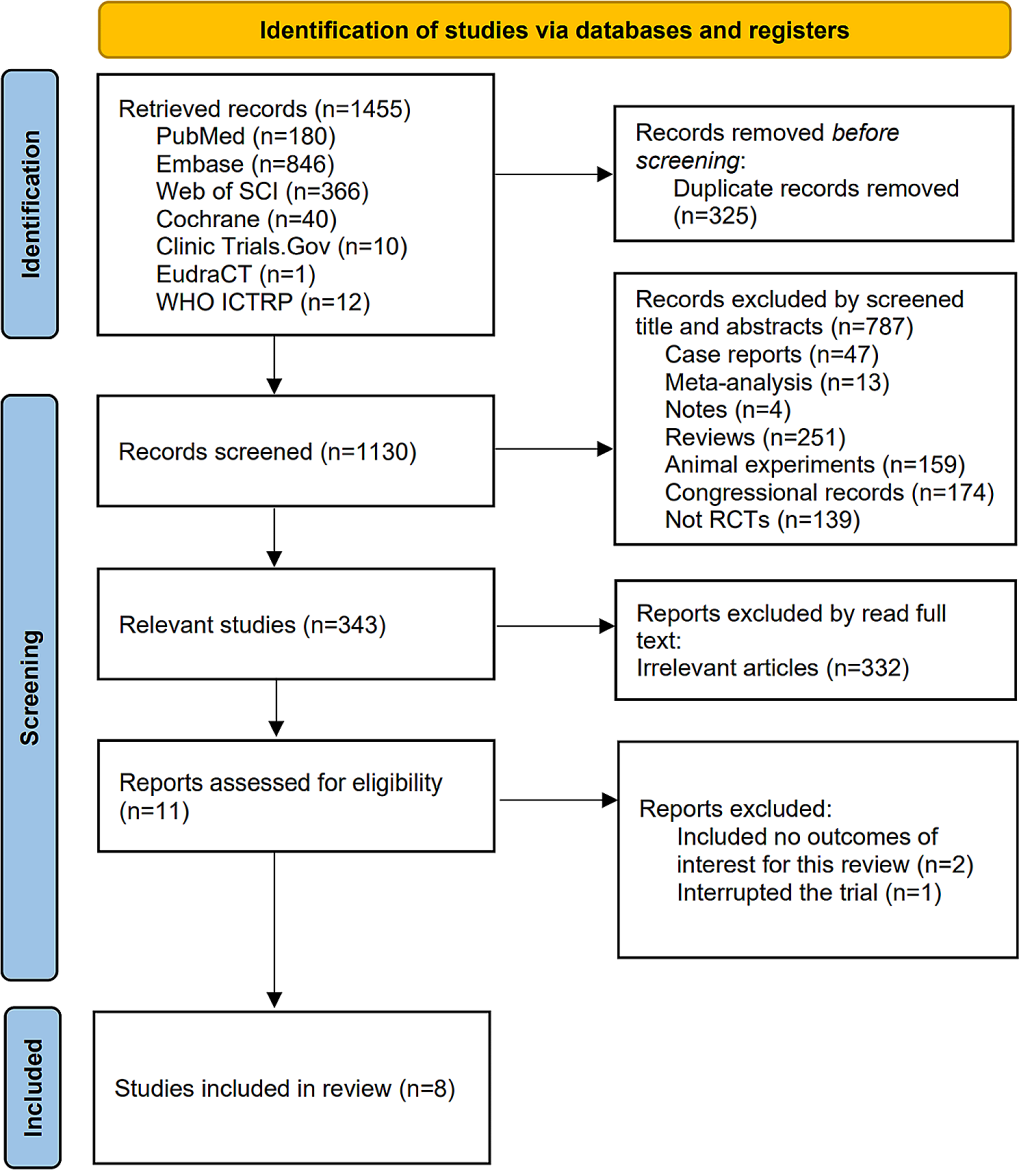
Flow diagram for the selection of the included studies

Figure 1 shows the PRISMA flowchart of the process and results of literature search. A thorough eligibility screening was conducted as per the PICOS (Population, Intervention, Comparison, Outcome, Study design) principle. A total of 1455 articles were retrieved from 7 databases. After removing duplicates (325 articles), a total of 1130 articles remained. Following the initial screening, 343 articles were obtained, out of which 332 irrelevant articles were excluded after full-text reading. We carefully read the remaining 11 articles [13, 23–32]. Among them, two studies [13, 23] were excluded because they did not report the outcomes of interest, and one study [24] was also excluded because the experiment was terminated prematurely. Finally, 8 studies [25–32] were eligible and included, and their quality was assessed.

### Basic information of included studies

The basic information of the included studies is presented in Table 1. A total of 8 articles were included. These articles were published between 1995 and 2023 and were all in English. The total number of eligible cases was 493, including 239 in the experimental group and 254 in the control group. Among these trials, two [26, 28] administered HBOT only once, while the remaining six trials had HBOT sessions of no less than seven times. One article [28] reported dropouts, and 5 articles [25–28, 30] conducted follow-ups with a duration not exceeding six months. The reported outcome measures included: (1) NIHSS score in five articles [25, 26, 29, 30, 32]; (2) Barthel index in two articles [26, 29]; (3) modified Rankin score in two articles [26, 27]; (4) TNF-α in two articles [29, 32]; (5) sICAM in two articles [26, 31]; (6) sVCAM in two articles [26, 31]; (7) sE-selectin in two articles [26, 31]; (8) CRP in two articles [29, 32]; and (9) mention of adverse reactions in four articles [27, 28, 30, 32]. Additionally, the HBOT intervention modes varied among the 8 studies, which could potentially affect the efficacy of HBOT. Therefore, we collected data on HBOT parameters, intervention duration, start time, etc., from each study, which are presented in Table 2.

**Table 1:** Characteristics of the included studies

Study	Patients(male)			Year(mean ± sd)			Intervention			Disease course(mean ± sd)		HBOT Efficacy	The CEBM Levels of Evidence
	IG	CG		IG	CG		IG	CG		IG	CG		
Chen 2012	16(13)	30(15)		68.375 ± NR	68.867 ± NR		HBOT + RDT + RT	RDT + RT		1.75 ± NR(day)	1.7 ± NR(day)	No difference (after 10 days) Beneficial (after 1 month)	Ib
Chen 2018	25(10)	25(11)		61.3 ± 8.7	62.7 ± 12.5		HBOT + APT	APT		NR	NR	Beneficial	IIb
Nighoghossian 1995	17(9)	17(12)		53 ± 3	54 ± 3		HBOT + RDT + RT	SHBOT + RDT + RT		19 ± 2.7(hour)	18 ± 3.2(hour)	Possibly beneficial	Ib
Rusyniak 2003	17(12)	16(10)		75 ± NR	68 ± NR		HBOT	SHBOT		NR	NR	No difference and Possibly harmful	Ib
Zhu 2022	55(40)	45(30)		63.12 ± 9.74	61.18 ± 8.31		HBOT + NWM	NWM		NR	NR	Beneficial	IIb
-Imai 2006	19(10)	19(12)		74.9 ± 12.1	73.7 ± 10.6		HBOT + IE + RDT	IE + RDT		546 ± 856(min)	668 ± 639(min)	Possibly beneficial	IIb
Zhao 2008	50(28)	62(36)		65.7 ± 12.9	68.2 ± 13.7		HBOT + RDT + RT	RDT + RT		NR	NR	Beneficial	IIb
Dong 2023	40(27)	40(25)		67.25 ± 3.51	66.80 ± 3.65		HBOT + NBP + OXR	NBP + OXR		6.81 ± 2.43(day)	7.10 ± 2.66(day)	Beneficial	IIb

IG: interventional group;CG: controlled group; NR: not reported; RDT: routine drug treatment; RT: rehabilitation therapy; APT: anti-platelet therapy; SHBOT: sham HBOT; NWM: needle-warming moxibustion; IE: intravenous edaravone; NBP: butylphthalide; OXR: oxiracetam

**Table 2:** The modes of HBOT intervention in the 8 RCTs

Study	FiO_2_	Pressure	Begin time	Treatment time	Therapy duration
Chen 2012	100%O_2_	2ATA	Within 3 ~ 5days after stroke onset	60 min	qd, 10days
Chen 2018	100%O_2_	2.5ATA	NR	60 min	Once
Nighoghossian 1995	100%O_2_/air^#^	1.5ATA/1.5ATA^**#**^	Within 24 h after stroke onset	40 min/40min^#^	a total of 10 dives
Rusyniak 2003	100%O_2_/100%O_2_^#^	2.5ATA/1.14ATA^**#**^	Within 24 h since symptom onset	60 min/60min^#^	Once
Zhu 2022	NR	2ATA	NR	80 min	q3d, 36days
Imai 2006	100%O_2_	2ATA	NR	60 min	qd, 1week
Zhao 2008	99%O_2_	2ATA	Within 1 ~ 3days following admission	60 min	qd, 10days
Dong 2023	NR	2ATA	NR	60 min	qd, 12weeks^*^

IG: interventional group;CG: controlled group; NR: not reported; qd: once a day; q3d: once every 3 days

^#^Data are given as Treatment/Control

^*^10 cycles as a treatment course, with an interval of three days between courses

### Quality assessment of included studies

**Fig. 2:**
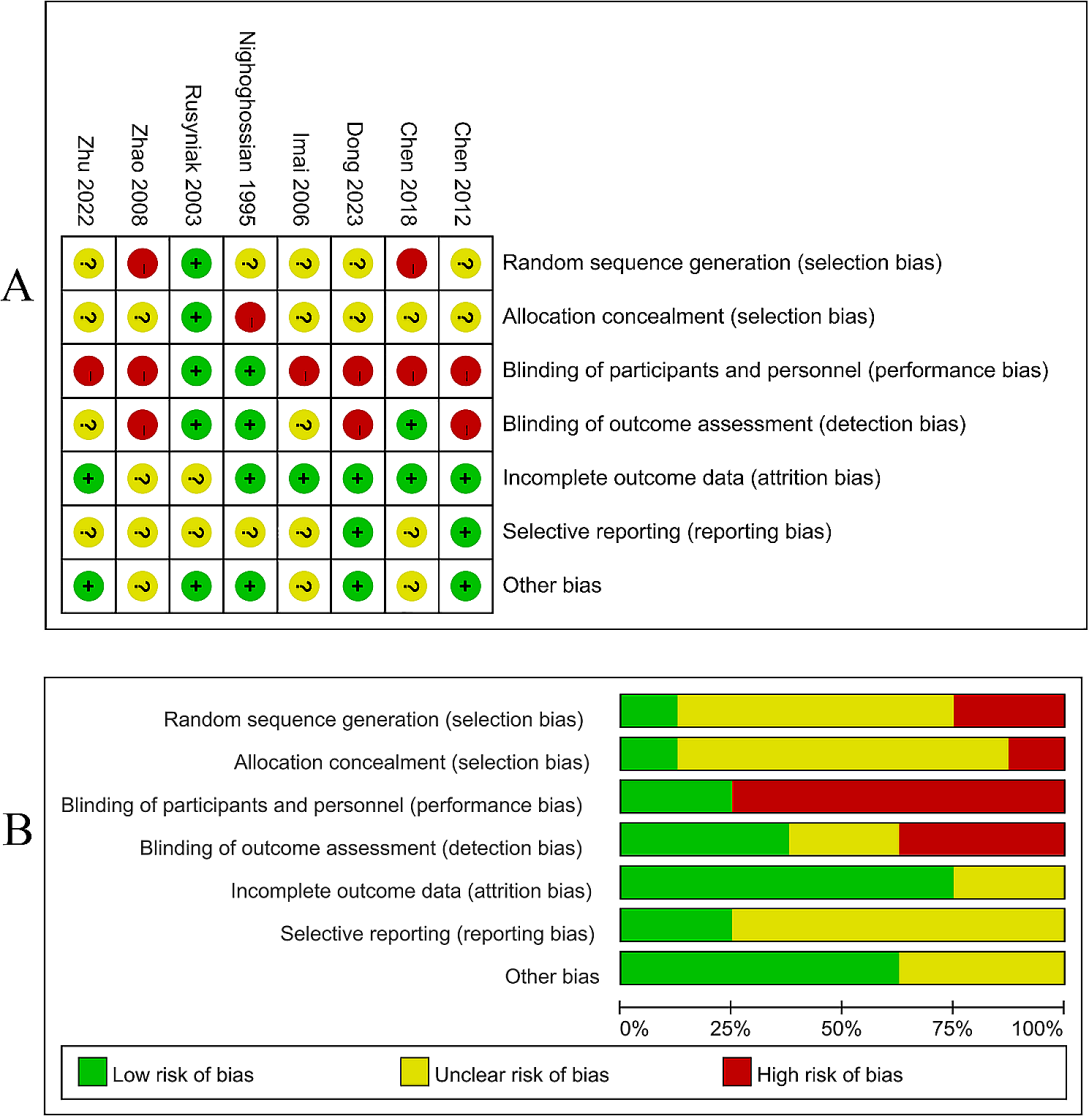
**(A)** Methodological quality summary; **(B)** Methodological quality graph

**Fig. 3:**
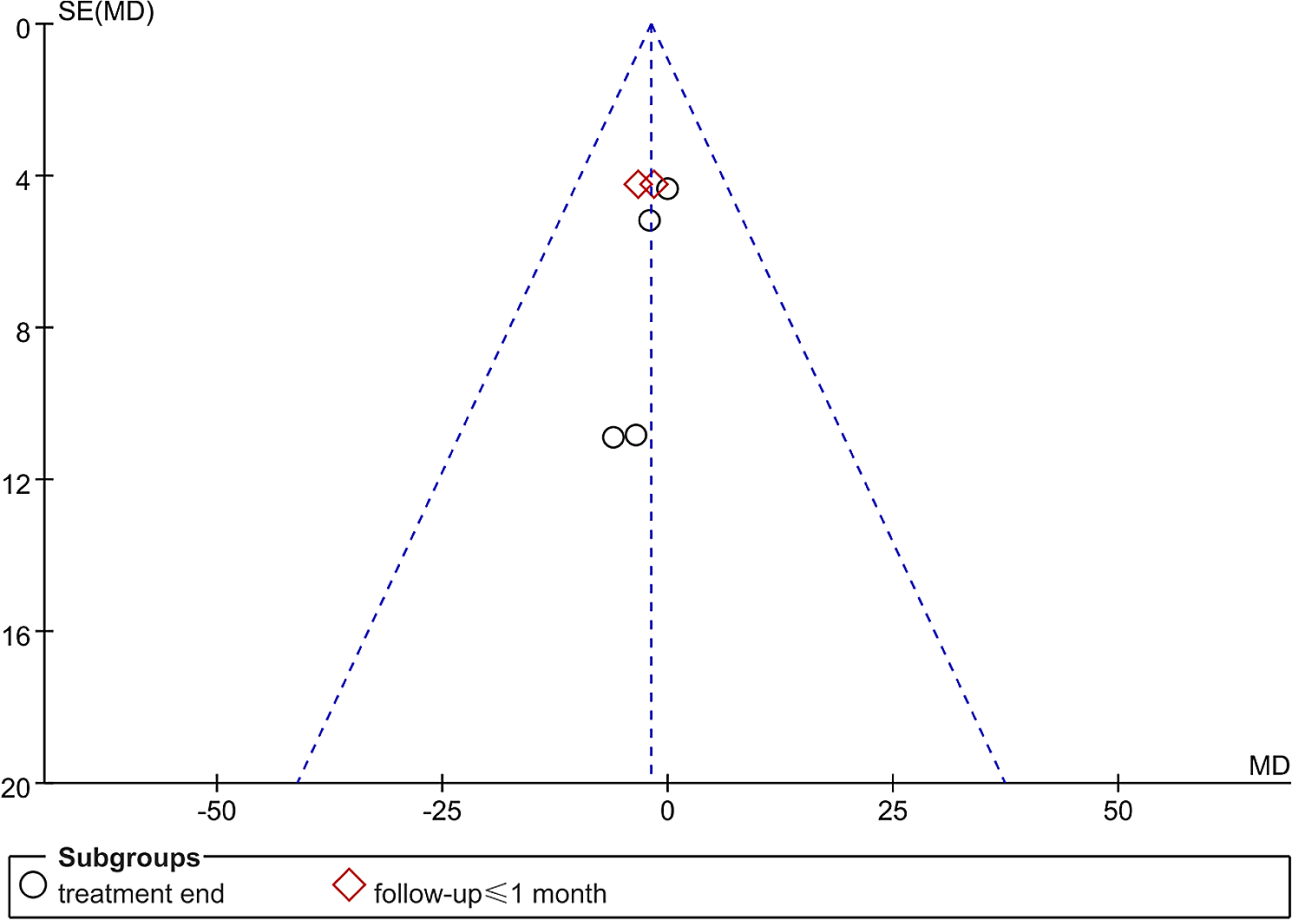
Publication bias from the involved studies

The quality assessment of the included studies is shown in Fig. 2A **and B**. Using the Cochrane Collaboration’s tool for assessing the risk of bias, we identified 7 studies [25–27, 29–32] with a high risk of bias. Furthermore, the Oxford Centre for Evidence-Based Medicine Levels of Evidence was utilized to ensure the quality of the included literature. In this scale, the scores for each study were 2b or higher, as listed in Table 1. Publication bias was assessed using a funnel plot, as depicted in Fig. 3. The symmetrical curve indicates low publication bias of these studies. A summary of the results for each article was conducted, and the outcomes of HBOT were categorized as “beneficial,” “possibly beneficial,” “no difference,” or “possibly harmful,” as listed in Table 1.

### Primary outcome measures

#### NIHSS score

**Fig. 4:**
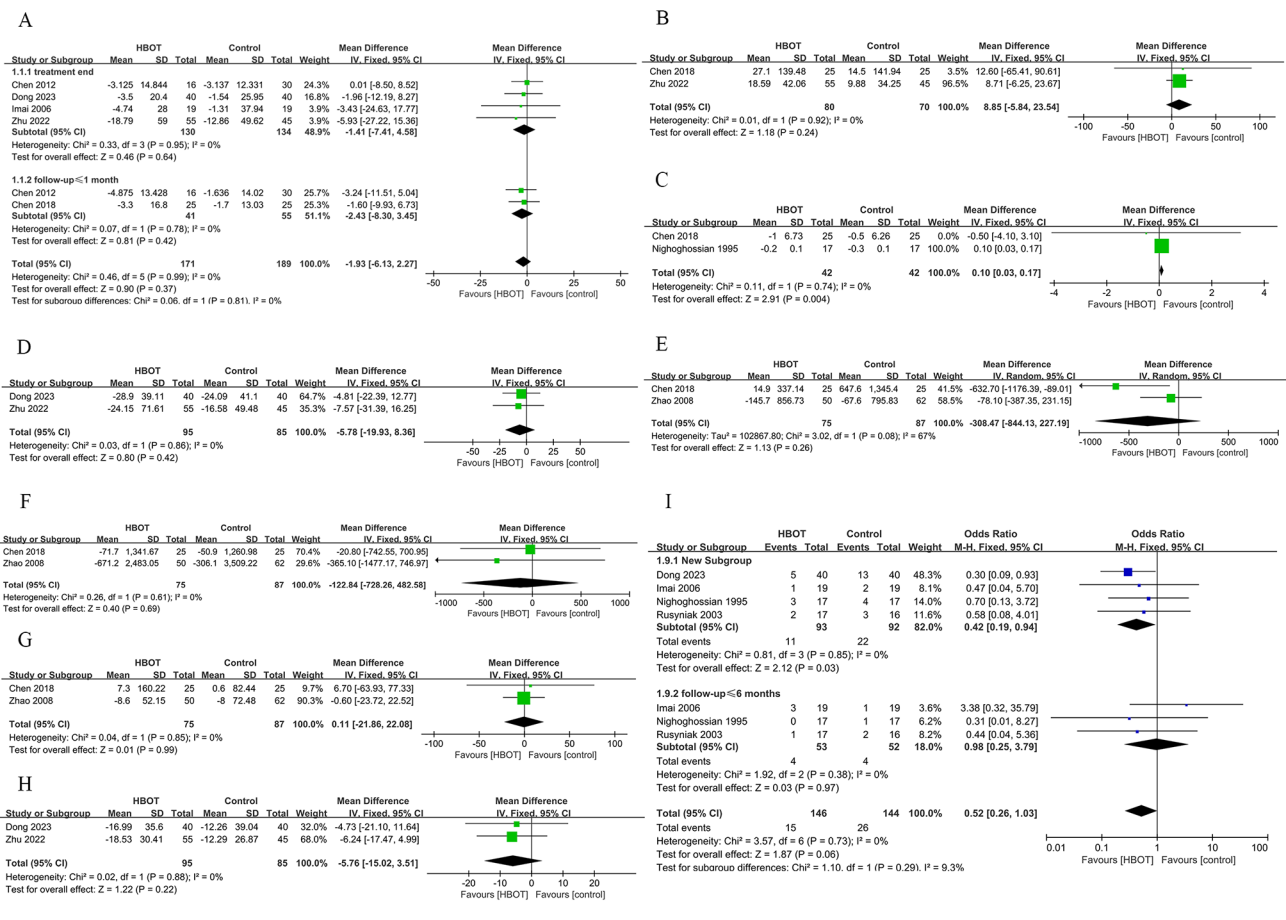
**(A)** The forest plot for NIHSS; **(B)** The forest plot for Barthel; **(C)** The forest plot for Modified Rankin; **(D)** The forest plot for TNF-α; **(E)** The forest plot for sICAM; **(F)** The forest plot for sVCAM; **(G)** The forest plot for sE-selectin; **(H)** The forest plot for CRP; **(I)** The forest plot for adverse events

Five articles reported the NIHSS score at the end of treatment, with 130 participants in the experimental group and 134 in the control group. Meta-analysis was performed on 4 RCTs using a fixed-effects model, as shown in Fig. 4A. The results indicated that although the HBOT group showed improvement, there was no statistically significant difference (MD = -1.41, 95%CI = -7.41 to 4.58, *P* = 0.64). Furthermore, two publications reported follow-up results with a follow-up duration of one month each. Subgroup analysis was performed, revealing no statistically significant difference in NIHSS scores (MD = -2.43, 95%CI = -8.30 to 3.45, *P* = 0.42).

#### Barthel index

Two articles reported the Barthel index, with 80 participants in the experimental group and 70 in the control group. Meta-analysis was performed on two RCTs using a fixed-effects model, as shown in Fig. 4B. The results indicated no statistically significant difference in HBOT scores between the HBOT group and the control group (MD = 8.85, 95%CI = -5.84 to 23.54, *P* = 0.24).

#### Modified rankin score

Two articles reported the modified Rankin score, with 42 participants in the experimental group and 42 in the control group. Meta-analysis was performed on two RCTs using a fixed-effects model, as shown in Fig. 4C. The results showed that the HBOT group had significantly better improvement in modified Rankin score compared to the control group (MD = 0.10, 95%CI = 0.03 to 0.17, *P* = 0.004).

#### Tumor necrosis factor-alpha (TNF-α)

Two articles reported TNF-α, with 95 participants in the experimental group and 85 in the control group. Meta-analysis was performed on three RCTs using a fixed-effects model, as shown in Fig. 4D. The results showed no statistically significant difference in TNF-α levels between the HBOT group and the control group (MD = -5.78, 95%CI = -19.93 to -8.36, *P* = 0.42).

#### Soluble intercellular adhesion molecule (sICAM)

Two articles reported sICAM, with 75 participants in the experimental group and 87 in the control group. Meta-analysis was performed on two RCTs using a random-effects model, as shown in Fig. 4E. The results revealed that although the HBOT group showed improvement in sICAM levels, there was no statistically significant difference (MD = -308.47, 95%CI = -844.13 to 13227.19, *P* = 0.26).

#### Soluble vascular cell adhesion molecule (sVCAM)

Two articles reported sVCAM, with 75 participants in the experimental group and 87 in the control group. Meta-analysis was performed on two RCTs using a fixed-effects model, as shown in Fig. 4F. The results revealed that although the HBOT group showed improvement in sVCAM levels, there was no statistically significant difference (MD = -122.84, 95%CI = -728.26 to 482.58, *P* = 0.69).

#### Soluble e-selectin (sE-selectin)

Two articles reported sE-selectin, with 75 participants in the experimental group and 87 in the control group. Meta-analysis was performed on two RCTs using a fixed-effects model, as shown in Fig. 4G. The results demonstrated that although the HBOT group exhibited improvement in sE-selectin levels, there was no statistically significant difference (MD = 0.11, 95%CI = -21.86 to 22.08, *P* = 0.99).

#### C-reactive protein (CRP)

Two articles reported CRP, with 95 participants in the experimental group and 85 in the control group. Meta-analysis was performed on two RCTs using a fixed-effects model, as shown in Fig. 4H. The results indicated that although the HBOT group showed improvement in CRP levels, there was no statistically significant difference (MD = -5.76, 95%CI = -15.02 to 3.51, *P* = 0.22).

### Secondary outcome measures

#### Adverse events

Four articles reported adverse events, with 93 participants in the experimental group and 92 in the control group. Adverse events included claustrophobia, fever, ear pain, and rash. Among them, three articles [24, 25, 28] reported serious adverse events such as heart failure and death. Meta-analysis was performed on four RCTs using a fixed-effects model, as shown in Fig. 4I. The results showed that the incidence of adverse events was significantly lower in the HBOT group compared to the control group (OR = 0.42, 95%CI = 0.19 to 0.94, *P* = 0.03). Additionally, three articles reported follow-up results, with a follow-up period not exceeding 6 months. Subgroup analysis revealed no significant difference between the groups in terms of adverse events, indicating that HBOT did not increase the incidence of adverse reactions (OR = 0.98, 95%CI = 0.25 to 3.79, *P* = 0.97).

## Discussion

Our study findings are based on 8 studies involving 493 patients with AIS. The results indicate that despite the inclusion of the latest research, the current evidence is insufficient to confirm a significant improvement in the prognosis of AIS with adjunctive HBOT.

Our literature review identified several previous systematic reviews and meta-analyses [12, 14, 20] on similar topics. Among them, Bennett MH published two studies evaluating the impact of HBOT on AIS outcomes [14, 20], with the latter being an update and supplement to the former. However, these studies by Bennett MH only summarized data on mortality cases. Due to the different scales used in each study, many data related to functional and quality of life assessments were not pooled and analyzed. In another study by Onose G, HBOT was included in the meta-analysis along with acupuncture, cooling therapy, and transcranial direct current stimulation, as one of the non-invasive, non-pharmacological intervention methods [12]. The author emphasized the impact of non-invasive, non-pharmacological interventions on AIS without thoroughly investigating the clinical effects of HBOT. Although our conclusions are similar to previous meta-analyses [14], we included a significant amount of previously unanalyzed data from scales and biomarkers. Furthermore, our study incorporated more recent clinical studies on HBOT for treating AIS. These factors contribute to the reliability and persuasiveness of our research.

We found that there were no statistically significant differences between the two groups in terms of NIHSS score, Barthel index, sICAM, TNF-α levels, sVCAM, sE-selectin, CRP, and incidence of adverse events within a follow-up period of ≤ 6 months. However, the HBOT group showed significantly better improvement in modified Rankin score, and adverse event incidences at the end of treatment compared to the control group. In contrast to previous studies [14], we found a significantly lower adverse event incidence in the HBOT group at the end of treatment. This suggests that HBOT may reduce adverse events in the short term. The short-term effects of HBOT have also been confirmed by Chen CY’s study [26]. However, some studies indicate that the benefits of high oxygen are not long-lasting and gradually diminish within 15 days after exposure to hyperoxia [33].

Our study specifically excluded interventions using thrombolysis and thrombectomy, the gold standard treatments for AIS recommended by American guidelines [8]. These methods, preferred for their effectiveness, are limited by strict time windows: thrombolysis to 4.5 h, benefiting only 3% of stroke patients [34]. Thrombectomy, effective for clots in larger brain arteries like the middle cerebral artery, offers a longer window. However, thrombectomy has more stringent requirements for preoperative assessment and surgical procedures. Complications such as vessel rupture, vasospasm, or thrombus dislodgment can exacerbate the condition. Due to the strict time windows and indications for thrombolysis and thrombectomy, patients may not benefit from these interventions. Therefore, other measures are needed to improve the prognosis of AIS. The purpose of HBOT is to increase the concentration of oxygen in the plasma to oxygenate ischemic and hypoxic tissues. Our study aimed to assess the impact of HBOT on AIS prognosis, measured by NIHSS scores, Barthel index, TNF-α, sICAM, sVCAM, sE-selectin, or CRP. While our findings do not support the use of HBOT for improving AIS prognosis, we hypothesize that this might be influenced by the extent of cerebral occlusion. An animal study using ischemic/reperfusion injury models shows that HBOT significantly reduces lipid peroxidation in the cerebral cortex and striatum [35]. Conversely, in a permanent middle cerebral artery occlusion (MCAO) model, HBOT does not significantly reduce lipid peroxidation levels [36]. The inconsistent results of HBOT between brains with MCAO and those with partial reperfusion also extend to oxidative stress. Oxidative stress refers to the rapid increase in harmful reactive oxygen and nitrogen species after ischemic stroke, damaging brain tissue [37]. In ischemic/reperfusion injury models, animal studies show HBOT significantly reduces malondialdehyde (MDA) and nitric oxide (NO) levels, indicators of oxidative stress severity [38]. However, in the MCAO model, NO levels increased [39]. These results suggest that HBOT’s effectiveness might depend on the extent of blood flow restoration. In brains with complete occlusion, we expect HBOT to rescue the ischemic penumbra by increasing blood oxygen concentration. In partially reperfused brains, we hope it can reduce lipid peroxidation and oxidative stress. While our current data from these eight RCTs are not sufficient to support this hypothesis, our study highlights the potential of HBOT to improve AIS and provides valuable insights for future research directions, making it nonetheless valuable.

HBOT for ischemic stroke has three main stages: pre-treatment, early treatment (acute phase), and neurofunctional recovery (chronic phase). However, the use of HBOT in the acute phase remains highly controversial [14]. AIS can be divided into two stages: the acute phase and the subacute phase [40]. Our analysis of four clinical trials supports this, as those [27, 28] initiating HBOT within 0–24 h (acute phase) reported no improvement, while the three studies [25, 31] starting HBOT within 1–5 days (subacute phase) observed positive clinical outcomes. This suggests that HBOT may be more effective in the subacute phase than the acute phase of AIS. However, some studies suggest HBOT may be relevant in the acute phase within a specific window. A study by Badr et al. [16, 17] showed HBOT applied within 6 h of ischemia reperfusion injury effectively reduce infarct size and improve neurological function, indicating potential benefits for patients. However, limited research on the early acute phase hinders definitive conclusions. Further high-quality studies are needed to address this critical gap in knowledge.

One trial conducted by Anderson DC et al. [24], which was previously included in the meta-analysis by Bennett MH et al., was not included in our study. This is because we discovered that Anderson DC’s trial allowed patients to withdraw from the study before completing 15 sessions of treatment. Out of 39 patients, 27 terminated the trial prematurely, citing reasons such as discharge, financial constraints, or patient refusal to continue treatment. This may introduce a significant risk of bias, so it was excluded.

In addition, we aimed to explore whether adjunctive HBOT can improve AIS prognosis in non-thrombolysis/thrombectomy treatment strategies. Our inclusion criteria were informed by previous research [12, 20] focusing on HBOT combined with rehabilitation methods alongside conventional medical care. We believe these rehabilitation methods, even though not considered the gold standard like thrombolysis/thrombectomy, deserve further evaluation for their potential benefit in AIS management. Clinically, such non-pharmacological treatments like oxygen therapy, acupuncture, and electrical stimulation are often combined with conventional medical care. One included article even used HBOT combined with acupuncture. Acupuncture is widely used in stroke treatment and recommended by the World Health Organization for its practicality and potential benefits [41]. In fact, growing evidence suggests these non-invasive non-drug methods hold promise for stroke treatment [11]. Therefore, we investigated whether adding HBOT to such combined interventions further improves AIS rehabilitation. Despite variations in interventions across studies, our study remains meaningful.

However, our systematic review still has some limitations including potential bias in seven studies [25–27, 29–32]. For instance, the Chen CY et al. [26] study, where treatment choices were based on patient preferences, could affect the reliability of the findings. Additionally, limited data for some outcome measures (reported in only 2–3 studies) suggest insufficient data for robust conclusions. Finally, due to a limited number of studies reporting the initiation timing of HBOT and varied outcome measures, subgroup analysis based on initiation time is not performed.

Building on prior meta-analyses, we reaffirm HBOT’s safety as an intervention. While our study doesn’t provide conclusive evidence for improved clinical outcomes in AIS [2], it highlights the need for further research exploring HBOT’s optimal timing and effectiveness across varying degrees of cerebral blockage. HBOT’s potential benefits cannot be ignored, and our findings pave the way for future research that could significantly impact AIS treatment strategies.

## Conclusion

While our study doesn’t provide conclusive evidence to significantly enhance clinical outcomes with adjunctive HBOT, it reaffirms its safety in treating AIS. While the potential for clinical benefit remains compelling, further research exploring HBOT’s optimal timing and effectiveness in different stroke scenarios is crucial.

### Electronic supplementary material

Below is the link to the electronic supplementary material.

Supplementary Material 1

## Data Availability

All data generated or analyzed during this study are included in this published article (and its Supplementary Information files).
