# Supplementary material for: Efficacy and safety of hyperbaric oxygen therapy in acute ischaemic stroke: a systematic review and meta-analysis
Source: BMC Neurol. 2024 Feb 3;24:55. doi: 10.1186/s12883-024-03555-w (PMC10837997; doi:10.1186/s12883-024-03555-w)
Supplement: Supplementary file 1 — Supplementary Material 1 [file 12883_2024_3555_MOESM1_ESM.docx]

**Appendix 1 Search strategy**

1. PubMed Search

| **NO** | **Search Details** | **Results** |
| --- | --- | --- |
| #1 | "Hyperbaric Oxygenation"[MeSH Terms] | 12,660 |
| #2 | "Ischemic Stroke"[MeSH Terms] | 9,859 |
| #3 | "hyperbaric oxygenation"[MeSH Terms] OR ("hyperbaric"[All Fields] AND "oxygenation"[All Fields]) OR "hyperbaric oxygenation"[All Fields] OR ("hyperbaric"[All Fields] AND "oxygenations"[All Fields]) OR "hyperbaric oxygenations"[All Fields] OR ("hyperbaric oxygenation"[MeSH Terms] OR ("hyperbaric"[All Fields] AND "oxygenation"[All Fields]) OR "hyperbaric oxygenation"[All Fields] OR ("hyperbaric"[All Fields] AND "oxygen"[All Fields] AND "therapy"[All Fields]) OR "hyperbaric oxygen therapy"[All Fields]) OR ("hyperbaric oxygenation"[MeSH Terms] OR ("hyperbaric"[All Fields] AND "oxygenation"[All Fields]) OR "hyperbaric oxygenation"[All Fields] OR ("hyperbaric"[All Fields] AND "oxygen"[All Fields] AND "therapies"[All Fields]) OR "hyperbaric oxygen therapies"[All Fields]) OR ("hyperbaric oxygenation"[MeSH Terms] OR ("hyperbaric"[All Fields] AND "oxygenation"[All Fields]) OR "hyperbaric oxygenation"[All Fields]) | 15,236 |
| #4 | "ischemic stroke"[MeSH Terms] OR ("ischemic"[All Fields] AND "stroke"[All Fields]) OR "ischemic stroke"[All Fields] OR ("ischemic"[All Fields] AND "strokes"[All Fields]) OR "ischemic strokes"[All Fields] OR ("ischemic stroke"[MeSH Terms] OR ("ischemic"[All Fields] AND "stroke"[All Fields]) OR "ischemic stroke"[All Fields] OR ("stroke"[All Fields] AND "ischemic"[All Fields]) OR "stroke ischemic"[All Fields]) OR ("ischemic stroke"[MeSH Terms] OR ("ischemic"[All Fields] AND "stroke"[All Fields]) OR "ischemic stroke"[All Fields] OR ("ischaemic"[All Fields] AND "stroke"[All Fields]) OR "ischaemic stroke"[All Fields]) OR ("ischemic stroke"[MeSH Terms] OR ("ischemic"[All Fields] AND "stroke"[All Fields]) OR "ischemic stroke"[All Fields] OR ("ischaemic"[All Fields] AND "strokes"[All Fields]) OR "ischaemic strokes"[All Fields]) OR ("ischemic stroke"[MeSH Terms] OR ("ischemic"[All Fields] AND "stroke"[All Fields]) OR "ischemic stroke"[All Fields] OR ("stroke"[All Fields] AND "ischaemic"[All Fields]) OR "stroke ischaemic"[All Fields]) OR ("ischemic stroke"[MeSH Terms] OR ("ischemic"[All Fields] AND "stroke"[All Fields]) OR "ischemic stroke"[All Fields] OR ("cryptogenic"[All Fields] AND "ischemic"[All Fields] AND "stroke"[All Fields]) OR "cryptogenic ischemic stroke"[All Fields]) OR ("ischemic stroke"[MeSH Terms] OR ("ischemic"[All Fields] AND "stroke"[All Fields]) OR "ischemic stroke"[All Fields] OR ("cryptogenic"[All Fields] AND "ischemic"[All Fields] AND "strokes"[All Fields]) OR "cryptogenic ischemic strokes"[All Fields]) OR ("ischemic stroke"[MeSH Terms] OR ("ischemic"[All Fields] AND "stroke"[All Fields]) OR "ischemic stroke"[All Fields] OR ("ischemic"[All Fields] AND "stroke"[All Fields] AND "cryptogenic"[All Fields]) OR "ischemic stroke cryptogenic"[All Fields]) OR ("ischemic stroke"[MeSH Terms] OR ("ischemic"[All Fields] AND "stroke"[All Fields]) OR "ischemic stroke"[All Fields] OR ("stroke"[All Fields] AND "cryptogenic"[All Fields] AND "ischemic"[All Fields])) OR ("ischemic stroke"[MeSH Terms] OR ("ischemic"[All Fields] AND "stroke"[All Fields]) OR "ischemic stroke"[All Fields] OR ("cryptogenic"[All Fields] AND "stroke"[All Fields]) OR "cryptogenic stroke"[All Fields]) OR ("ischemic stroke"[MeSH Terms] OR ("ischemic"[All Fields] AND "stroke"[All Fields]) OR "ischemic stroke"[All Fields] OR ("cryptogenic"[All Fields] AND "strokes"[All Fields]) OR "cryptogenic strokes"[All Fields]) OR ("ischemic stroke"[MeSH Terms] OR ("ischemic"[All Fields] AND "stroke"[All Fields]) OR "ischemic stroke"[All Fields] OR ("stroke"[All Fields] AND "cryptogenic"[All Fields]) OR "stroke cryptogenic"[All Fields]) OR ("ischemic stroke"[MeSH Terms] OR ("ischemic"[All Fields] AND "stroke"[All Fields]) OR "ischemic stroke"[All Fields] OR ("cryptogenic"[All Fields] AND "embolism"[All Fields] AND "stroke"[All Fields]) OR "cryptogenic embolism stroke"[All Fields]) OR ("ischemic stroke"[MeSH Terms] OR ("ischemic"[All Fields] AND "stroke"[All Fields]) OR "ischemic stroke"[All Fields] OR ("cryptogenic"[All Fields] AND "embolism"[All Fields] AND "strokes"[All Fields])) OR ("ischemic stroke"[MeSH Terms] OR ("ischemic"[All Fields] AND "stroke"[All Fields]) OR "ischemic stroke"[All Fields] OR ("embolism"[All Fields] AND "stroke"[All Fields] AND "cryptogenic"[All Fields])) OR ("ischemic stroke"[MeSH Terms] OR ("ischemic"[All Fields] AND "stroke"[All Fields]) OR "ischemic stroke"[All Fields] OR ("stroke"[All Fields] AND "cryptogenic"[All Fields] AND "embolism"[All Fields])) OR ("ischemic stroke"[MeSH Terms] OR ("ischemic"[All Fields] AND "stroke"[All Fields]) OR "ischemic stroke"[All Fields] OR ("wake"[All Fields] AND "up"[All Fields] AND "stroke"[All Fields]) OR "wake up stroke"[All Fields]) OR ("ischemic stroke"[MeSH Terms] OR ("ischemic"[All Fields] AND "stroke"[All Fields]) OR "ischemic stroke"[All Fields] OR ("stroke"[All Fields] AND "wake"[All Fields] AND "up"[All Fields]) OR "stroke wake up"[All Fields]) OR ("ischemic stroke"[MeSH Terms] OR ("ischemic"[All Fields] AND "stroke"[All Fields]) OR "ischemic stroke"[All Fields] OR ("wake"[All Fields] AND "up"[All Fields] AND "stroke"[All Fields]) OR "wake up stroke"[All Fields]) OR ("ischemic stroke"[MeSH Terms] OR ("ischemic"[All Fields] AND "stroke"[All Fields]) OR "ischemic stroke"[All Fields] OR ("wake"[All Fields] AND "up"[All Fields] AND "strokes"[All Fields]) OR "wake up strokes"[All Fields]) OR ("ischemic stroke"[MeSH Terms] OR ("ischemic"[All Fields] AND "stroke"[All Fields]) OR "ischemic stroke"[All Fields] OR ("acute"[All Fields] AND "ischemic"[All Fields] AND "stroke"[All Fields]) OR "acute ischemic stroke"[All Fields]) OR ("ischemic stroke"[MeSH Terms] OR ("ischemic"[All Fields] AND "stroke"[All Fields]) OR "ischemic stroke"[All Fields] OR ("acute"[All Fields] AND "ischemic"[All Fields] AND "strokes"[All Fields]) OR "acute ischemic strokes"[All Fields]) OR ("ischemic stroke"[MeSH Terms] OR ("ischemic"[All Fields] AND "stroke"[All Fields]) OR "ischemic stroke"[All Fields] OR ("ischemic"[All Fields] AND "stroke"[All Fields] AND "acute"[All Fields]) OR "ischemic stroke acute"[All Fields]) OR ("ischemic stroke"[MeSH Terms] OR ("ischemic"[All Fields] AND "stroke"[All Fields]) OR "ischemic stroke"[All Fields] OR ("stroke"[All Fields] AND "acute"[All Fields] AND "ischemic"[All Fields]) OR "stroke acute ischemic"[All Fields]) | 113,546 |
| #5 | #1 or #3 | 15,236 |
| #6 | #2 or #4 | 113,546 |
| #7 | #5 and #6 | 180 |

2. EMbase Search

| **NO** | **Search Details** | **Results** |
| --- | --- | --- |
| #1 | 'hyperbaric oxygen therapy'/exp OR 'hyperbaric oxygen therapy' OR (hyperbaric AND ('oxygen'/exp OR oxygen) AND ('therapy'/exp OR therapy)) OR 'hbo therapy' OR (high AND pressure AND oxygen) OR (high AND tension AND o2) OR (high AND tension AND oxygen) OR (hyperbaric AND medicine) OR (hyperbaric AND o2) OR (hyperbaric AND oxygen) OR (hyperbaric AND oxygen AND treatment) OR (hyperbaric AND oxygenation) OR (hyperbaric AND oxygenisation) OR (hyperbaric AND oxygenization) OR (hyperbaric AND therapy) OR (oxygen, AND hyperbaric) | 73,991 |
| #2 | 'ischemic stroke'/exp OR 'ischemic stroke' OR (ischemic AND ('stroke'/exp OR stroke)) OR (ischaemic AND stroke) OR (ischemic AND strokes) OR (stroke, AND ischemic) OR (ischaemic AND strokes) OR (cryptogenic AND ischemic AND stroke) OR (cryptogenic AND ischemic AND strokes) OR (cryptogenic AND stroke) OR (cryptogenic AND strokes) OR (cryptogenic AND embolism AND stroke) OR (cryptogenic AND embolism AND strokes) OR ('wake up' AND stroke) OR (wake AND up AND stroke) OR ('wake up' AND strokes) OR (acute AND ischemic AND stroke) OR (acute AND ischemic AND strokes) | 212,489 |
| #3 | #1 AND #2 | 846 |

3. Web of Science Search

| **NO** | **Search Details** | **Results** |
| --- | --- | --- |
| #1 | "((((TS=(Hyperbaric Oxygenations)) OR TS=(Hyperbaric Oxygen Therapy)) OR TS=(Hyperbaric Oxygen Therapies)) OR TS=(Hyperbaric Oxygenation)) OR TS=(Hyperbaric Oxygenation)" | 18,859 |
| #2 | "((((((((((((((((((((((((TS=(ischaemic stroke)) OR TS=(Ischemic Strokes)) OR TS=(Stroke, Ischemic)) OR TS=(Ischaemic Stroke)) OR TS=(Ischaemic Strokes)) OR TS=(Stroke, Ischaemic)) OR TS=(Cryptogenic Ischemic Stroke)) OR TS=(Cryptogenic Ischemic Strokes)) OR TS=(Ischemic Stroke, Cryptogenic)) OR TS=(Stroke, Cryptogenic Ischemic)) OR TS=(Cryptogenic Stroke)) OR TS=(Cryptogenic Strokes)) OR TS=(Stroke, Cryptogenic)) OR TS=(Cryptogenic Embolism Stroke)) OR TS=(Cryptogenic Embolism Strokes)) OR TS=(Embolism Stroke, Cryptogenic)) OR TS=(Stroke, Cryptogenic Embolism)) OR TS=(Wake-up Stroke)) OR TS=(Stroke, Wake-up)) OR TS=(Wake up Stroke)) OR TS=(Wake-up Strokes)) OR TS=(Acute Ischemic Stroke)) OR TS=(Acute Ischemic Strokes)) OR TS=(Ischemic Stroke, Acute)) OR TS=(Stroke, Acute Ischemic)" | 174,625 |
| #3 | #1 AND #2 | 366 |

4. Cochrane Library Search

| **NO** | **Search Details** | **Results** |
| --- | --- | --- |
| #1 | MeSH descriptor: [Hyperbaric Oxygenation] explode all trees | 530 |
| #2 | MeSH descriptor: [lschemic Stroke] explode all trees | 862 |
| #3 | (Hyperbaric Oxygenations):ti,ab,kw OR (Hyperbaric Oxygen Therapy):ti,ab,kw OR (Hyperbaric Oxygen Therapies):ti,ab,kw OR (Hyperbaric Oxygenation):ti,ab,kw | 1,740 |
| #4 | (Ischemic Strokes):ti,ab,kw OR (Stroke, Ischemic):ti,ab,kw OR (lschaemic Stroke):ti,ab,kw OR (lschaemic Strokes):ti,ab,kw OR (Stroke, Ischaemic):ti,ab,kw | 18,325 |
| #5 | (Cryptogenic Ischemic Stroke):ti,ab,kw OR (Cryptogenic Ischemic Strokes):ti,ab,kw OR (Ischemic Stroke, Cryptogenic):ti,ab,kw OR (Stroke, Cryptogenic Ischemic):ti,ab,kw OR (Cryptogenic Stroke):ti,ab,kw | 264 |
| #6 | (Cryptogenic Strokes):ti,ab,kw OR (Stroke, Cryptogenic):ti,ab,kw OR (Cryptogenic Embolism Stroke):ti,ab,kw OR (Cryptogenic Embolism Strokes):ti,ab,kw OR (Embolism Stroke, Cryptogenic):ti,ab,kw | 264 |
| #7 | (Stroke, Cryptogenic Embolism):ti,ab,kw OR (Wake-up Stroke):ti,ab,kw OR (Stroke, Wake-up):ti,ab,kw OR (Wake up Stroke):ti,ab,kw OR (Wake-up Strokes):ti,ab,kw | 394 |
| #8 | (Acute Ischemic Stroke):ti,ab,kw OR (Acute Ischemic Strokes):ti,ab,kw OR (lschemic Stroke, Acute):ti,ab,kw OR (Stroke, Acute Ischemic):ti,ab,kw | 9,062 |
| #9 | #1 OR #3 | 1,740 |
| #10 | #2 OR #4 OR #5 OR #6 O R#7 OR #8 | 18,610 |
| #11 | #9 and #10 | 41 |

5. ClinicalTrials.Gov Search

| **NO** | **Search Details** | **Results** |
| --- | --- | --- |
| #1 | Hyperbaric oxygen \| Interventional Studies \| Stroke | 10 |

6. EudraCT Search

| **NO** | **Search Details** | **Results** |
| --- | --- | --- |
| #1 | Stroke AND hyperbaric oxygen | 1 |

7. WHO ICTRP Search

| NO | Search Details | Results |
| --- | --- | --- |
| #1 | Stroke AND hyperbaric oxygen | 12 |
